# The Role of Artificial Intelligence in the Primary Prevention of Common Musculoskeletal Diseases

**DOI:** 10.7759/cureus.65372

**Published:** 2024-07-25

**Authors:** Selkin Yilmaz Muluk, Nazli Olcucu

**Affiliations:** 1 Physical Medicine and Rehabilitation, Antalya City Hospital, Antalya, TUR; 2 Physical Medicine and Rehabilitation, Antalya Atatürk State Hospital, Antalya, TUR

**Keywords:** digital health, chatgpt, primary prevention, musculoskeletal disorders, artificial intelligence (ai)

## Abstract

Background: Musculoskeletal disorders (MSDs) are a leading cause of disability worldwide, with a growing burden across all demographics. With advancements in technology, conversational artificial intelligence (AI) platforms such as ChatGPT (OpenAI, San Francisco, CA) have become instrumental in disseminating health information. This study evaluated the effectiveness of ChatGPT versions 3.5 and 4 in delivering primary prevention information for common MSDs, emphasizing that the study is focused on prevention and not on diagnosis.

Methods: This mixed-methods study employed the CLEAR tool to assess the quality of responses from ChatGPT versions in terms of completeness, lack of false information, evidence support, appropriateness, and relevance. Responses were evaluated independently by two expert raters in a blinded manner. Statistical analyses included Wilcoxon signed-rank tests and paired samples t-tests to compare the performance across versions.

Results: ChatGPT-3.5 and ChatGPT-4 effectively provided primary prevention information, with overall performance ranging from satisfactory to excellent. Responses for low back pain, fractures, knee osteoarthritis, neck pain, and gout received excellent scores from both versions. Additionally, ChatGPT-4 was better than ChatGPT-3.5 in terms of completeness (p = 0.015), appropriateness (p = 0.007), and relevance (p = 0.036), and ChatGPT-4 performed better across most medical conditions (p = 0.010).

Conclusions: ChatGPT versions 3.5 and 4 are effective tools for disseminating primary prevention information for common MSDs, with ChatGPT-4 showing superior performance. This study underscores the potential of AI in enhancing public health strategies through reliable and accessible health communication. Advanced models such as ChatGPT-4 can effectively contribute to the primary prevention of MSDs by delivering high-quality health information, highlighting the role of AIs in addressing the global burden of chronic diseases. It is important to note that these AI tools are intended for preventive education purposes only and not for diagnostic use. Continuous improvements are necessary to fully harness the potential of AI in preventive medicine. Future studies should explore other AI platforms, languages, and secondary and tertiary prevention measures to maximize the utility of AIs in global health contexts.

## Introduction

Musculoskeletal disorders (MSDs) are conditions that affect motor organs such as muscles, tendons, bones, and nerves, causing discomfort or severe injuries. Common MSDs include diseases presenting with low back pain and neck pain, osteoarthritis, gout, and rheumatoid arthritis. In 2019, approximately 323 million new cases of MSDs were reported globally, resulting in significant disability and death. The majority of incident cases were low back pain (67.15%), followed by neck pain (14.28%), osteoarthritis (12.46%), gout (2.77%), and rheumatoid arthritis (0.32%). Low back pain contributed the most to disability-adjusted life years (42.44%), followed by neck pain (14.71%), osteoarthritis (12.63%), rheumatoid arthritis (2.17%), and gout (1.12%) [1].

In 2021, MSDs presenting with low back pain have become the leading cause of years lived with disability, accounting for 7.7% of all causes. Other MSDs ranked fifth, accounting for 4.8% of all causes, with osteoarthritis and neck pain also ranked 14th and 15th, respectively [2]. The prevalence of MSDs is increasing globally across all sexes and ages [3]. These data underline the urgent need for targeted interventions to prevent MSDs.

Primary prevention is a fundamental public health concept that focuses on preventing diseases or injuries before they occur. This approach aims to reduce the incidence of diseases by addressing risk factors and implementing strategies to maintain health and well-being. Health education and active patient engagement can be a practical aspect of it.

Conversational artificial intelligence (AI) platforms have emerged as valuable tools for providing health information. These platforms leverage large language models to provide rapid responses that mimic human speech. They have made tremendous progress in the health field [4]. AI in medicine includes virtual AI (such as electronic health records and conversational AI) and physical AI (such as surgical robots and intelligent prostheses), revolutionizing modern medicine by enhancing clinical diagnosis, drug discovery, and patient care. AI systems analyze extensive medical data, including images and genomic information, to detect patterns and provide insights that might be challenging for clinicians. Conversational AIs can be used in various medical areas such as imaging analysis, clinical diagnosis, drug discovery, patient support, remote monitoring, surgical assistance, personalized treatment plans, administrative tasks, medical documentation, and patient education [5].

Given their success in various medical fields, conversational AIs can also be used for primary prevention of diseases. A notable example is ChatGPT, developed by OpenAI (San Francisco, CA) [6]. This study evaluated the effectiveness of ChatGPT versions 3.5 and 4 in providing health information related to the primary prevention of common MSDs. We assessed their responses for completeness, lack of false information, evidence support, appropriateness, and relevance.

The objective of this study is to evaluate the effectiveness of ChatGPT versions 3.5 and 4 in delivering primary prevention information for common MSDs, emphasizing that the study is focused on primary prevention and not on diagnosis.

Our research was driven by two hypotheses. The effectiveness hypothesis posits that both versions of ChatGPT will effectively answer questions related to the primary prevention of common MSDs. The null hypothesis for this is that neither version will effectively answer the questions. The performance hypothesis suggests that ChatGPT-4 will demonstrate superior performance compared to ChatGPT-3.5 due to its more advanced training and capabilities. The null hypothesis for this is that there will be no difference in performance between ChatGPT-3.5 and ChatGPT-4.

These hypotheses underscore the importance of validating the utility of AI tools in managing the growing health burden of MSD, reflecting a broader initiative to integrate AI effectively into primary prevention strategies.

## Materials and methods

Study design

This study combined qualitative and quantitative elements, making it a mixed-methods study. The study was prepared according to the METRICS checklist specifically prepared to design and report AI-based studies, which involve M (model used and its exact settings), E (evaluation approach for the generated content), T (timing of testing the model, transparency of the data source), R (range of tested topics, randomization of selecting the queries), I (individual factors in selecting the queries and interrater reliability), C (count of queries executed to test the model), and S (specificity of the prompts and language used) [7].

As the study did not involve the direct participation of human subjects and was primarily focused on interactions with conversational AI systems, formal ethical approval was not sought or required.

Models used and their exact settings

We selected ChatGPT, developed by OpenAI, as it was among the most popular conversational AIs available during the search period. The versions used were exemplary representations of modern conversational AI systems. ChatGPT-3.5 was freely accessible to the public, and ChatGPT-4 required a subscription for access.

Both systems were evaluated using standard default settings to ensure that the produced content could be replicated. As ChatGPT-3.5 and ChatGPT-4 do not retain information from previous interactions, and as each new conversation started fresh, without access to past queries or responses, potential learning or feedback loops were accepted to be prevented. Nevertheless, all questions were asked using a new chat button, and the regenerate button was not used.

Evaluation approach for the generated content

An assistant posed the preformed questions to the ChatGPT versions, collected their responses, and anonymized the data by assigning fake names to them. The authors then assessed the responses, remaining unaware of the versions' identities and blinded to each other's scoring results.

The evaluation of responses was based on the CLEAR tool. The items of this tool include C (completeness of content), L (lack of false information in the content), E (evidence supporting the content), A (appropriateness of the content), and R (relevance). In this tool, each item was scored as follows: excellent = 5, very good = 4, good = 3, satisfactory/fair = 2, or poor = 1. Although the CLEAR tool requires further validation and comparison with established tools such as DISCERN, CDC Clear Communication Index, and Patient Education Materials Assessment Tool, it was chosen for its specific advantages and value in assessing AI-generated health information [8].

Timing of testing the models and transparency of the data source

Testing of both versions of ChatGPT was conducted on February 21, 2024, at local time 9.30-10.30, in the Istanbul zone. Conversations were documented in the public data repository Zenodo with doi: 10.5281/zenodo.11978306 [9].

Range of tested topics and randomization of selecting the queries

We carefully prepared a comprehensive set of questions to cover all of the most common MSDs. Due to our systematic approach to question selection, randomization was not required for this study. This method allowed us to cover the full spectrum of commonly encountered scenarios by including all the related diseases.

Individual factors in selecting the queries and interrater reliability

Medline and Google Scholar searches were conducted using the keywords "musculoskeletal diseases," "prevalence," and "epidemiology" for the period from 2013 to 2023. Among the associated data, we incorporated pertinent information from reputable global epidemiological studies and databases, including Global Burden of Disease studies and related analyses [1-3,10]. These sources helped us formulate the research questions. By selecting the 15 most common MSDs, we minimized selection bias, ensuring that no individual factors influenced query selection.

The content produced by ChatGPT versions was evaluated independently by two authors, designated as rater 1 and rater 2. Both raters were specialist physiatrists working in outpatient clinics and physical medicine and rehabilitation services. Cohen's kappa statistic was used to evaluate the agreement between the two raters and to measure interrater reliability.

Count of queries executed to test the model

We formulated 15 queries corresponding to the 15 MSDs. They were designed to test the ChatGPT versions' responses. The exact queries posed to the AI are presented in Table 1.

**Table 1:** Queries addressed to ChatGPT-3.5 and ChatGPT-4 about primary prevention of common musculoskeletal diseases AI: artificial intelligence

	Preventive health queries addressed to AI
1	What preventive measures and lifestyle changes can I implement to reduce my risk of developing low back pain? I'm seeking guidance on primary prevention strategies for this condition.
2	What preventive measures and lifestyle changes can I implement to reduce my risk of developing a fracture? I'm seeking guidance on primary prevention strategies for this condition.
3	What preventive measures and lifestyle changes can I implement to reduce my risk of developing knee osteoarthritis? I'm seeking guidance on primary prevention strategies for this condition.
4	What preventive measures and lifestyle changes can I implement to reduce my risk of developing hand osteoarthritis? I'm seeking guidance on primary prevention strategies for this condition.
5	What preventive measures and lifestyle changes can I implement to reduce my risk of developing hip osteoarthritis? I'm seeking guidance on primary prevention strategies for this condition.
6	What preventive measures and lifestyle changes can I implement to reduce my risk of developing neck pain? I'm seeking guidance on primary prevention strategies for this condition.
7	What preventive measures and lifestyle changes can I implement to reduce my risk of developing extremity amputation? I'm seeking guidance on primary prevention strategies for this condition.
8	What preventive measures and lifestyle changes can I implement to reduce my risk of developing gout? I'm seeking guidance on primary prevention strategies for this condition.
9	What preventive measures and lifestyle changes can I implement to reduce my risk of developing rheumatoid arthritis? I'm seeking guidance on primary prevention strategies for this condition.
10	What preventive measures and lifestyle changes can I implement to reduce my risk of developing rotator cuff muscle strain? I'm seeking guidance on primary prevention strategies for this condition.
11	What preventive measures and lifestyle changes can I implement to reduce my risk of developing ankle sprain? I'm seeking guidance on primary prevention strategies for this condition.
12	What preventive measures and lifestyle changes can I implement to reduce my risk of developing psoriatic arthritis? I'm seeking guidance on primary prevention strategies for this condition.
13	What preventive measures and lifestyle changes can I implement to reduce my risk of developing spondyloarthropathy? I'm seeking guidance on primary prevention strategies for this condition.
14	What preventive measures and lifestyle changes can I implement to reduce my risk of developing fibromyalgia? I'm seeking guidance on primary prevention strategies for this condition.
15	What preventive measures and lifestyle changes can I implement to reduce my risk of developing systemic lupus erythematosus? I'm seeking guidance on primary prevention strategies for this condition.

Specificity of the prompts and language used

Each inquiry adhered to a uniform method, initiating with a precise introductory phrase "What preventive measures and lifestyle changes can I implement to reduce my risk of developing …?" and ending with a concluding query: "I'm seeking guidance on primary prevention strategies for this condition." We intentionally prepared our questions in a manner that mimicked the queries of real persons using plain language to address health-related concerns. This approach aimed to capture the authenticity and natural tone of humans seeking medical information. In doing so, we strived to ensure that our interactions with the AI system closely resembled real-world scenarios, thereby making the results and recommendations more relatable to the public. All the inquiries were conducted in English.

Statistics and data analysis

Statistical analysis was conducted using IBM SPSS Statistics for Windows version 29.0.2.0 (IBM Corp., Armonk, NY). The level of statistical significance was set at p < 0.050.

The agreement between two independent raters was measured using Cohen's kappa statistic. Cohen's kappa values were categorized as follows: values less than 0.20 indicated poor agreement, 0.21-0.40 fair agreement, 0.41-0.60 moderate agreement, 0.61-0.80 substantial agreement, and 0.81-1.00 almost perfect agreement [11].

After assessing interrater reliability, the scores for each item from the two raters were summed and then divided by two. The results were referred to as "average CLEAR scores." For example, the C score from rater 1 and the C score from rater 2 were summed and then divided by two, with the resultant value interpreted as the "average C score." These average scores indicated the quality of the generated content for each item.

Additionally, the average scores of the five items of the tool (C, L, E, A, and R) were later summed and divided by five. The results were referred to as "overall CLEAR scores," indicating the overall quality of the generated content. The classification of the overall CLEAR scores was as follows: CLEAR scores of 1-1.79 were classified as "poor," 1.80-2.59 as "satisfactory," 2.60-3.39 as "good," 3.40-4.19 as "very good," and 4.20-5.00 as "excellent" [8].

The Wilcoxon signed-rank test was used to compare the average scores of ChatGPT-3.5 and ChatGPT-4 for each CLEAR item. This non-parametric test was chosen because the CLEAR scores are ordinal in nature, and the test does not assume a normal distribution of the differences between paired observations. The Wilcoxon signed-rank test is suitable for comparing two related samples, making it ideal for our study where the same items were rated for both versions of ChatGPT.

For comparing the overall scores of ChatGPT-3.5 and ChatGPT-4 for each medical condition, a paired samples t-test was used. Before applying this test, the normality of the distribution of the data was assessed using the Kolmogorov-Smirnov and Shapiro-Wilk tests. The paired samples t-test was chosen because it is appropriate for comparing the means of two related groups and assumes that the differences between the paired observations are normally distributed. This test allowed us to evaluate if there was a statistically significant difference in the overall performance of the two ChatGPT versions across various medical conditions.

For further analysis to assess performance per CLEAR item, we examined the within-model variability in performance.

## Results

In this study, we evaluated the effectiveness of ChatGPT versions 3.5 and 4 in delivering primary prevention information for common MSDs. Both versions demonstrated notable proficiency in offering relevant advices. Table 2 summarizes the primary prevention recommendations that were commonly provided by both versions, as well as unique suggestions provided by each version.

**Table 2:** Comparison of the responses received from ChatGPT versions Vit D: vitamin D, DM: diabetes mellitus, RA: rheumatoid arthritis, CVD: cardiovascular disease, PAD: peripheral artery disease, BP: blood pressure, PsA: psoriatic arthritis, SLE: systemic lupus erythematosus

MSD	ChatGPT-3.5 recommendations	ChatGPT-4 recommendations	Common recommendations
Low back pain	Staying hydrated	Regular checkups	Regular exercise and physical activity; healthy weight; proper posture, sleeping position, lifting, and footwear; ergonomic workstations; managing stress; quitting smoking
Fractures	Fall risk assessment by a professional	Protein intake; limiting sodium	Regular exercise and checkups; fall prevention; medication and supplements/calcium and Vit D; sun exposure; avoiding smoking and alcohol; bone density measurement; healthy weight
Knee osteoarthritis	Management of existing health conditions such as DM, metabolic syndrome, and RA	Glucosamine and chondroitin supplements; staying hydrated	Healthy weight; regular exercise; proper diet, posture, and footwear; avoiding injury; protecting joints; regular checkups
Hand osteoarthritis	Proper posture; managing stress	Managing chronic conditions such as DM and CVD; regular checkups	Healthy weight; regular exercise; proper diet; avoiding overuse; protecting joints; staying hydrated; quitting smoking
Hip osteoarthritis	Proper body mechanics; managing stress	Regular checkups; limiting alcohol; glucosamine and chondroitin supplements; staying hydrated	Healthy weight; regular exercise; proper diet and posture; avoiding overuse; protecting joints; avoiding smoking
Neck pain	Proper lifting	Avoiding injury	Proper posture, ergonomics, and sleeping positions; regular exercise and physical activity; taking frequent breaks in work; avoidance of prolonged smartphone use; managing stress; healthy weight; staying hydrated
Extremity amputation	Protecting extremities; adhering to medications	Proper diet; limiting alcohol	Healthy blood sugar levels and weight; lifestyle to manage PAD/heart-healthy lifestyle; regular checkups; proper foot care; seeking prompt treatment for foot injuries and infections; regular exercise; quitting smoking; managing BP and cholesterol levels; educating yourself about DM and PAD
Gout	Quitting smoking	Considering uric acid-lowering medications; monitoring BP, cholesterol, and blood sugar levels	Healthy weight; limiting foods rich in purine, alcohol, and diuretics; limiting sugary foods and beverages; staying hydrated; regular exercise; avoiding rapid weight loss and crash diets; managing stress
Rheumatoid arthritis	Prioritizing sleep; protecting joints; monitoring joints for swelling and pain	Minimizing exposure to environmental pollutants such as silica and asbestos; monitoring and managing chronic inflammation such as periodontal diseases; genetic counseling	Healthy weight; anti-inflammatory diet; regular exercise; avoiding smoking and alcohol; managing stress; regular checkups
Rotator cuff muscle strain	Staying hydrated; cross-training to prevent overuse; using proper equipment in sports	Ergonomic workstations; limiting overhead activities; quitting smoking; regular checkups	Warming up before physical activity; strengthening and flexibility exercises; proper posture; proper technique in shoulder activities/proper alignment of the shoulder; regular breaks/adequate rest; healthy weight; paying attention to signs of discomfort
Ankle sprain	Proper technique in jumping, pivoting, and changing direction	Proprioception training; diet rich in calcium and Vit D	Warming up before physical activity; strengthening and flexibility exercises; balance training; proper footwear; gradual progression in exercise intensity and duration; avoiding risks when walking on uneven terrain; healthy weight; using protective gear if there is a history of sprain
Psoriatic arthritis	Protecting the skin; informing healthcare provider if there is a family history of PsA	Protecting the joints; proper Vit D levels	Healthy weight; anti-inflammatory diet; regular exercise; managing stress; quitting smoking; limiting alcohol; regular checkups; treatment adherence
Spondyloarthropathy	Limiting alcohol	-	Healthy weight; regular exercise; proper posture; ergonomic furniture; quitting smoking; managing stress; regular checkups; anti-inflammatory diet; avoiding stress to joints
Fibromyalgia	-	Regular checkups; ergonomic work and home environments	Regular exercise; healthy weight; managing stress; cognitive behavioral therapy; improving sleep quality; limiting caffeine and alcohol; anti-inflammatory diet; staying hydrated; taking breaks in activities; support network with friends, family and healthcare professionals
Systemic lupus erythematosus	Healthy weight	Limit exposure to silica dust and pesticides; awareness of medications that can trigger SLE-like symptoms	Anti-inflammatory diet; regular exercise; sun protection; managing stress; avoiding smoking; limiting alcohol; regular checkups; informing healthcare provider if there is a family history of autoimmune disease

After the raters assessed these responses, the agreement between them was calculated. Cohen's κ values ranged from 0.524 to 0.885, indicating moderate to almost perfect agreement. This demonstrated reliable assessments of AI outputs across different CLEAR items in our study. ChatGPT-3.5 received average scores from the two raters between 3.7 and 4.2 across different CLEAR items, while ChatGPT-4 received scores ranging from 3.8 to 4.6. The lowest scores of both versions were for evidence support. The highest score of ChatGPT-3 was for relevance, and the highest scores of ChatGPT-4 were for appropriateness and relevance (Table 3).

**Table 3:** Assessment of interrater reliability for evaluating the outputs of ChatGPT-3.5 and ChatGPT-4, according to each CLEAR tool C: completeness, L: lack of false information, E: evidence support, A: appropriateness, R: relevance, SD: standard deviation, *: moderate agreement, **: substantial agreement, ***: almost perfect agreement Level of statistical significance for p value: <0.050

Scoring items	Rater 1	Rater 2	Average scores	Cohen's κ	Asymptotic standard error	Approximate T	p value
ChatGPT-3.5	Mean±SD	Mean±SD	Mean±SD				
C	3.9±0.704	4.2±0.561	4.1±0.594	0.535*	0.188	3.054	0.002
L	4.1±0.704	4.1±0.640	4.1±0.660	0.885***	0.110	4.670	<0.001
E	3.7±0.488	3.6±0.507	3.7±0.372	0.857***	0.137	3.354	<0.001
A	4.0±0.926	4.3±0.594	4.1±0.719	0.685**	0.147	3.980	<0.001
R	4.3±0.704	4.2±0.676	4.2±0.651	0.667**	0.179	3.392	<0.001
ChatGPT-4							
C	4.4±0.632	4.5±0.640	4.5±0.581	0.524*	0.174	2.480	0.013
L	4.5±0.516	4.4±0.736	4.5±0.500	0.535*	0.171	2.618	0.009
E	3.8±0.414	3.7±0.458	3.8±0.309	0.815***	0.176	3.211	0.001
A	4.7±0.488	4.7±0.488	4.6±0.481	0.700**	0.196	2.711	0.007
R	4.7±0.458	4.5±0.640	4.6±0.516	0.575*	0.190	2.557	0.011

**Figure 1:**
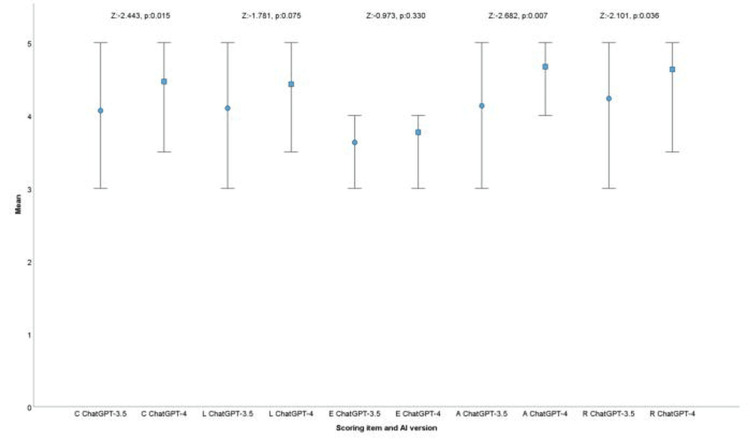
Comparison of average CLEAR scores for ChatGPT-3.5 and ChatGPT-4 per each CLEAR item Differences were assessed using the Wilcoxon signed-rank test, highlighting specific items where the scores significantly differ between versions, as indicated by the Z values and p values. C: completeness, L: lack of false information, E: evidence support, A: appropriateness, R: relevance Circles represent ChatGPT-3.5, and squares represent ChatGPT-4.

To compare the average CLEAR scores of ChatGPT versions for each item, a Wilcoxon signed-rank test was conducted. The results indicated significant differences in specific items: "completeness" showed a significant difference, with ChatGPT-4 performing better (p = 0.015). "Lack of false information" did not show a significant difference between ChatGPT-3.5 and ChatGPT-4 (p = 0.075). "Evidence support" also did not show a significant difference between the two versions (p = 0.330). "Appropriateness" showed a significant difference, with ChatGPT-4 performing better (p = 0.007). Finally, "relevance" showed a significant difference, again with ChatGPT-4 performing better (p = 0.036) (Figure 1).

When we used the overall CLEAR scores for each medical condition, we found that ChatGPT-4 generally performed better across most common MSDs. For instance, ChatGPT-4 received excellent ratings for all of the conditions except hand osteoarthritis, extremity amputation, and spondyloarthropathy, whereas ChatGPT-3.5 had lower ratings ranging from satisfactory to good for most of the conditions (Table 4).

**Table 4:** Comparison of overall CLEAR scores between ChatGPT-3.5 and ChatGPT-4 for each chronic medical condition The overall performance improvement was assessed using a paired samples t-test, indicating statistically significant differences (p < 0.050*). MSD: musculoskeletal disorder

MSD	Overall CLEAR score ChatGPT-3.5	Overall CLEAR score ChatGPT-4
Low back pain	4.8 (excellent)	4.8 (excellent)
Fractures	4.4 (excellent)	4.4 (excellent)
Knee osteoarthritis	4.4 (excellent)	4.3 (excellent)
Hand osteoarthritis	3.1 (satisfactory)	3.9 (good)
Hip osteoarthritis	3.9 (good)	4.8 (excellent)
Neck pain	4.8 (excellent)	4.2 (excellent)
Extremity amputation	3.1 (satisfactory)	3.5 (good)
Gout	4.8 (excellent)	4.8 (excellent)
Rheumatoid arthritis	3.8 (good)	4.6 (excellent)
Rotator cuff muscle strain	3.5 (good)	4.5 (excellent)
Ankle sprain	4.0 (good)	4.6 (excellent)
Psoriatic arthritis	3.9 (good)	4.3 (excellent)
Spondyloarthropathy	4.0 (good)	3.9 (good)
Fibromyalgia	4.0 (good)	4.8 (excellent)
Systemic lupus erythematosus	4.0 (good)	4.5 (excellent)
Overall statistical analysis	t value(14): -2.965	p value: 0.010*

To compare the overall CLEAR scores of ChatGPT versions, a paired samples t-test was conducted. The test yielded a t value of -2.965 with a significant p value of 0.010. This result indicated a statistically significant difference in performances between ChatGPT-3.5 and ChatGPT-4, with ChatGPT-4 showing improvements in most areas (Table 5).

**Table 5:** Paired samples t-test results comparing the overall CLEAR scores per query between ChatGPT-3.5 and ChatGPT-4 Both Kolmogorov-Smirnov and Shapiro-Wilk tests revealed a normal distribution of the data before the use of the t-test. SD: standard deviation, SEM: standard error mean, CI: confidence interval, df: degrees of freedom p value is two-sided and is considered statistically significant (p < 0.050*).

Paired differences	Mean	SD	SEM	95% CI	t	df	p
Overall CLEAR scores	-0.36000	0.47026	0.12142	-0.62042 to -0.09958	-2.965	14	0.010*

For further analysis to assess the performance per CLEAR item, we used within-model variability in performance. Upon comparing the performance of ChatGPT-3.5 across the CLEAR items, it was found that the model exhibited variability in its performance. However, these differences did not reach statistical significance for most items, with the exception of "evidence." Specifically, as the differences in "completeness," "lack of false information," "appropriateness," and "relevance" were not statistically significant, the performance in these areas was consistent. ChatGPT-4 also demonstrated variability in its performance across the CLEAR items. The chi-square tests confirmed significant differences in performance for "evidence" and "relevance." Conversely, the performance differences in "completeness," "lack of false information," and "appropriateness" did not reach statistical significance, indicating consistent performance in these areas (Table 6).

**Table 6:** Performance comparison across CLEAR items for ChatGPT-3.5 and ChatGPT-4 *: level of statistical significance for p value: <0.050

Item	ChatGPT-3.5 chi-square test	p value	ChatGPT-4 chi-square test	p value
Completeness	χ²(4) = 8.000	0.092	χ²(3) = 4.467	0.215
Lack of false information	χ²(3) = 7.667	0.053	χ²(3) = 5.533	0.137
Evidence	χ²(2) = 6.400	0.041*	χ²(2) = 11.200	0.004*
Appropriateness	χ²(3) = 3.400	0.334	χ²(2) = 5.200	0.074
Relevance	χ²(3) = 2.333	0.506	χ²(3) = 10.333	0.016*

## Discussion

Primary prevention in healthcare focuses on preventing the onset of diseases before they occur, an approach often overshadowed by the treatment of existing conditions. Despite its potential to significantly reduce healthcare costs and alleviate the burden on healthcare systems, the implementation of primary prevention remains low [12]. Our study aimed to evaluate the efficacy of ChatGPT versions 3.5 and 4 in delivering health information pertinent to the primary prevention of the most common 15 MSDs. AI systems have the potential to provide reliable information and create new opportunities for patient engagement, which is crucial for effective primary prevention approaches.

To our knowledge, this is the first study to evaluate the effectiveness of conversational AI in providing health information for the primary prevention of MSDs. This study provides valuable groundwork for future advancements in preventive medicine and health information systems, not only for MSDs but also for related fields.

In this study, both versions generally provided responses ranging from satisfactory to excellent. For example, ChatGPT-4 delivered excellent responses for 12 out of 15 common chronic MSDs, except for hand osteoarthritis, extremity amputation, and spondyloarthropathy. In contrast, ChatGPT-3.5 received excellent ratings for only five out of 15 conditions. Both models excelled in responding to questions about low back pain, fractures, knee osteoarthritis, neck pain, and gout. Despite the high performance of both models, ChatGPT-4 consistently outperformed ChatGPT-3.5 in several key areas, including completeness (p = 0.015), appropriateness (p = 0.007), and relevance (p = 0.036). ChatGPT-4 also performed better across most medical conditions (p = 0.010). The superior performance of ChatGPT-4 highlights advancements in AI capabilities, suggesting its enhanced utility in the primary prevention of diseases.

Our findings were consistent with our initial hypotheses. The results confirm our effectiveness hypothesis, demonstrating that both versions of ChatGPT can effectively disseminate crucial health information to empower individuals toward proactive health behaviors. Additionally, the enhanced performance of ChatGPT-4 supports our performance hypothesis, showing that its advanced training and algorithmic refinements lead to better handling of health questions.

Similar studies have been conducted on the use of conversational AIs, specifically ChatGPT, to evaluate health information regarding disease prevention. For example, one study found that 84% of the cardiovascular disease prevention advice provided by ChatGPT was appropriate for patient education and clinician communication [13]. Another study assessed ChatGPT responses to breast cancer prevention and screening, with 88% of the AI-generated responses deemed appropriate by fellowship-trained breast radiologists [14]. Additionally, a study evaluated ChatGPT-3.5's ability to respond accurately to questions about osteoporotic fracture prevention and medical science, with both patients and experts rating the responses as appropriate and comparable to those of medical professionals [15]. Another study highlighted ChatGPT's potential in suicide prevention, offering helpful responses, but emphasized the need for professional oversight, since ChatGPT is not a substitute for professional medical advice [16]. A study on ChatGPT's responses to HIV prevention questions found high accuracy and completeness but noted issues such as outdated terms and lack of representation for some communities [17].

In our study, both ChatGPT-3.5 and ChatGPT-4 generally demonstrated high performance in responding to primary prevention queries for common MSDs, aligning with the findings of similar studies. ChatGPT-3.5's responses were mostly relevant, while ChatGPT-4's responses were mostly appropriate and relevant. However, despite the absence of any incorrect information that could pose a risk to users' health, some responses were rated as satisfactory or good, rather than excellent. Additionally, the "evidence support" item received the lowest scores in both versions. Therefore, we also believe that ChatGPT is not yet a substitute for professional medical advice, and an expert touch remains essential to ensure optimal primary prevention of MSDs.

When reviewing the comparative studies in the literature on this topic, we found several relevant studies. A study assessed ChatGPT-3.5 and GPT-4 recommendations for prostate cancer prevention and screening and found that ChatGPT-4 outperformed its predecessor in terms of accuracy, clarity, conciseness, and readability [18]. Another study compared the effectiveness of ChatGPT-4 and ChatGPT-3.5 in assessing suicide risk, revealing that ChatGPT-4 closely matched mental health professionals' evaluations and more accurately identified suicidal ideation, whereas ChatGPT-3.5 tended to underestimate the risk, especially in severe cases [19]. Additionally, a study comparing the accuracy, comprehensibility, and response length of ChatGPT-3.5 and ChatGPT-4 for patient education on hyperlipidemia found that both versions provided accurate information; however, ChatGPT-4 offered more concise and readable responses, demonstrating superior adaptability and readability [20]. Furthermore, a study comparing the responses of five large language models, including ChatGPT-3.5 and ChatGPT-4, to common migraine-related queries found that ChatGPT-4 had the highest accuracy (96.7%). Despite some inaccuracies and the need for ongoing evaluation, this study underscores the potential of large language models, particularly ChatGPT-4, to enhance migraine patient education [21]. In our study, ChatGPT-4 outperformed ChatGPT-3.5 in average CLEAR scores, showing significant improvements in "completeness," "appropriateness," and "relevance." Overall, ChatGPT-4 performed better across medical conditions, with a statistically significant difference.

Building on these findings, the broader role of conversational AI in public health presents both benefits and challenges. When used for public health issues, ChatGPT offers strengths such as personalized health information, 24/7 access, and enhanced disease surveillance. However, it has weaknesses, such as misinterpretation risks, privacy breaches, and perpetuating data biases. Opportunities include providing personalized health support, especially to underserved populations, and improving provider-patient communication and care quality. Threats involve the distribution of incorrect information, replacement of human interaction, and worsening of the digital divide [22].

The practical implications of integrating ChatGPT-4 into healthcare practice are significant. Healthcare providers can use ChatGPT-4 as a supplementary tool to provide patients with reliable health information and reinforce primary prevention strategies. For instance, ChatGPT-4 can offer educational resources, answer patient queries outside of office hours, and provide reminders for preventive measures, thus enhancing patient engagement and adherence to health recommendations. However, potential barriers to its adoption include concerns about the accuracy and reliability of AI-generated information, the need for ongoing updates and validation, data privacy issues, and the possibility of reduced face-to-face interactions between patients and healthcare providers. Facilitators for successful integration include ensuring robust validation processes, continuous training of AI models, addressing data privacy concerns through stringent regulations, and combining AI tools with professional oversight to maintain high standards of care. By overcoming these barriers and leveraging facilitators, ChatGPT-4 can become a valuable asset in clinical settings, supporting healthcare providers in delivering comprehensive and accessible preventive care.

This study contributes to the growing body of literature on the efficacy of AI tools in public health. It aligns with the ideas presented above, demonstrating both the potential benefits and the challenges of integrating AI into healthcare. By evaluating the performance of ChatGPT versions in providing reliable health information, our research underscores the need for improvement of platforms and professional oversight.

Several limitations warrant careful consideration. The evaluations were subjective and descriptive; however, the high kappa values indicate a strong agreement among raters, reflecting their shared expertise. The questions were not randomized but were designed to cover all relevant diseases comprehensively. The small sample size of only 15 queries focused on the most common MSDs. Additionally, the study assessed only two versions of a conversational AI platform and was limited to the English language, which may limit the generalizability of the findings to non-English-speaking populations. Moreover, it only involved primary prevention queries.

Despite these limitations, the research remains valuable for the primary prevention of commonly encountered MSDs. It shows the important role that AI can play in enhancing primary prevention strategies, highlighting the need for broader research and integration to fully leverage AIs' capabilities in public health.

## Conclusions

This study provides evidence that conversational AI, particularly advanced models such as ChatGPT-4, can effectively contribute to the primary prevention of MSDs by delivering high-quality health information. This capability underscores the role of AIs in enhancing public health strategies and addressing the global burden of chronic diseases. However, improvement is necessary to fully harness the benefits of AIs in preventive medicine. The practical implications of integrating conversational AIs into healthcare are significant, as they can supplement patient education and preventive care. However, key barriers such as accuracy, validation, and privacy concerns need to be addressed, along with the need for ongoing updates and professional oversight.

Future studies should focus on increasing the number of questions, exploring more versions of the same platform, and including other AI platforms. Additionally, research should extend to languages other than English and cover secondary and tertiary prevention measures to maximize the utility of AI in diverse global health contexts. Specifically, studies should investigate AI's role in early disease detection, management, and reducing disease recurrence. Research should also explore the integration of AI tools with existing healthcare systems to streamline workflows and improve patient outcomes. These efforts will help establish comprehensive AI-driven strategies for disease prevention. By addressing these challenges and leveraging the strengths of AI, we can significantly advance the effectiveness of preventive healthcare worldwide. It is important to note that while AI tools such as ChatGPT can provide valuable information on primary prevention measures, they are not intended to be used as stand-alone diagnostic tools. Users are strongly encouraged to consult with healthcare professionals for accurate diagnosis and appropriate treatment of MSDs.
